# Amoebicidal Effect of COVID Box Molecules against *Acanthamoeba*: A Study of Cell Death

**DOI:** 10.3390/ph17060808

**Published:** 2024-06-20

**Authors:** Ines Sifaoui, Rubén L. Rodríguez-Expósito, María Reyes-Batlle, Robert Sutak, José E. Piñero, Jacob Lorenzo-Morales

**Affiliations:** 1 Instituto Universitario de Enfermedades Tropicales y Salud Pública de Canarias (IUETSPC), Universidad de La Laguna (ULL), Avda. Astrofísico Fco. Sánchez, S/N, 38200 San Cristóbal de La Laguna, Spain; isifaoui@ull.edu.es (I.S.); rrodrige@ull.edu.es (R.L.R.-E.); mreyesba@ull.edu.es (M.R.-B.); 2 Departamento de Obstetricia y Ginecología, Pediatría, Medicina Preventiva y Salud Pública, Toxicología, Medicina Legal y Forense y Parasitología, Universidad de La Laguna, 38200 San Cristóbal de La Laguna, Spain; 3 Consorcio Centro de Investigación on Biomédica En Red (CIBER), Área de Enfermedades Infecciosas (CIBERINFEC), Instituto de Salud Carlos III, 28220 Madrid, Spain; 4 Department of Parasitology, Faculty of Science, Charles University, BIOCEV, 252 50 Vestec, Czech Republic; robert.sutak@natur.cuni.cz

**Keywords:** COVID box, *Acanthamoeba*, amoebicidal activity, action mode, programmed cell death

## Abstract

*Acanthamoeba* spp. can cause a sight threatening disease. At present, the current treatments used to treat *Acanthamoeba* spp. Infections, such as biguanide-based antimicrobials, remain inefficacious, with the appearance of resistant forms and high cytotoxicity to host cells. In this study, an initial screening was conducted against *Acanthamoeba castellanii* Neff and murine macrophages J774A.1 using alamarBlue™. Among the 160 compounds included in the cited box, 90% exhibited an inhibition of the parasite above 80%, while only 18.75% of the compounds inhibited the parasite with a lethality towards murine macrophage lower than 20%. Based on the amoebicidal activity, the cytotoxicity assay, and availability, Terconazole was chosen for the elucidation of the action mode in two clinical strains, *Acanthamoeba culbertsoni* and *Acanthamoeba castellanii* L10. A fluorescence image-based system and proteomic techniques were used to investigate the effect of the present azole on the cytoskeleton network and various programmed cell death features, including chromatin condensation and mitochondria dysfunction. Taking all the results together, we can suggest that Terconazole can induce programmed cell death (PCD) via the inhibition of sterol biosynthesis inhibition.

## 1. Introduction

Free-living amoeba (FLA) are ubiquitous protozoa that have been isolated from natural and artificial habitats such as water, soil, and air conditioning systems, among others. Among the reported FLA, *Acanthamoeba* was the most isolated genus from both environmental and clinical samples. This amphizoid protozoan parasite presents two live stages: an actively feeding dividing trophozoite and a resistant inactive cyst [1]. Depending on the target tissue, *Acanthamoeba* spp. induces various diseases: granulomatous amoebic encephalitis (GAE) when they infect the central nerve system, *Acanthamoeba* Keratitis (AK) when they infect the eye, and cutaneous lesions when they attack the skin [2]. 

Recently, an increase in *Acanthamoeba* infections, mostly AK cases, has been reported. This outcome could be the result of increased awareness, the handiness of diagnostic tools, and the extensive effort in characterizing the isolated *Acanthamoeba* species [3]. Depending on the type of infection, the actual treatment includes biguanides and aromatic diamidines for AK, and pentamidine, sulfadiazine, flucytosine, and either fluconazole or itraconazole for the granulomatous encephalitis [4]. Yet these treatments are not fully and uniformly effective against the trophozoite and cyst forms of *Acanthamoeba* genus. Moreover, due to long-term drug administration, these treatments result in post-therapy problems of drug resistance, side effects, and toxicity [5].

Recently, after the COVID-19 pandemic, drug repositioning has gained great attention as an efficient and promising strategy for drug discovery compared to the traditional strategy [6]. In this sense, the “Medicines for Malaria Venture” (MMV) is considered a pioneer in the drug repurposing strategy by proposing their malaria-designed box for further diseases. The organization “Medicines for Malaria Venture” (MMV) has compiled libraries of various drugs known to exhibit in vitro anti-plasmodium activity and made them available for research all over the world to test them against other pathogens [7]. Until now, four open access boxes have been launched: the Malaria Box, the Pathogen Box, the Pandemic Response Box and the COVID Box [8]. Over 200 research group have evaluated the boxes against various diseases such as cancer and microbial infections, including *Leishmania*, *Acanthamoeba*, and *Toxoplasma gondii* [5,9,10,11]. The last box has been tested to discover a potent drug against the coronavirus and other pathogens like *Toxoplasma gondii* and *Naegleria fowleri* [12,13].

In previous works, we evaluated the activity of the MMV Pathogen Box library against *Acanthamoeba castellanii* Neff and *Leishmania amazonensis* [5,9]. Among the 400 molecules tested, two well-known molecules, Pentamidine and Posaconazole, were the most active against *Acanthamoeba castellanii* Neff [5], while in *Leishmania amazonensis*, five molecules were newly reported to inhibit the parasite by inducing programmed cell death (PCD) [9].

The aims of the present study were first to evaluate the amoebicidal activity of the COVID Box, a MMV collection of 160 molecules with recognized or predicted activity against the coronavirus SARS-CoV-2. Subsequently, a PCD study and proteomic analysis of the effect of the most active compound were conducted.

## 2. Results

### 2.1. In Vitro Assay of COVID Box Molecules against Acanthamoeba castellanii Neff and Murine Macrophages

**Figure 1:**
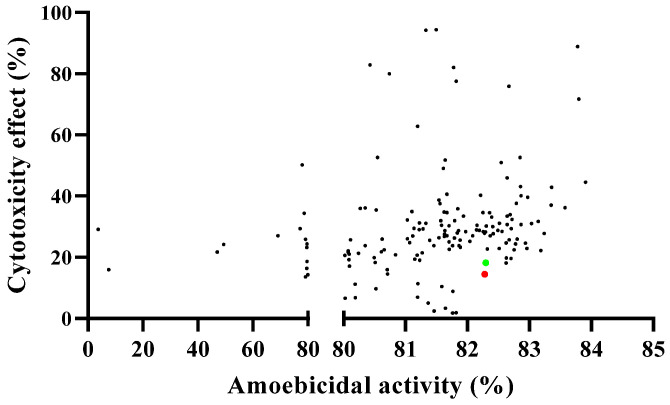
Descriptive analysis of the initial screening based on the trophocidal activity against *Acanthamoeba castellanii* Neff and the cytotoxicity effect against murine macrophage. The red spot represents Terconazole, while the green spot represents Almitrine.

The initial amoebicidal screening of the COVID Box molecules was conducted at 10 µM against the type of strain *Acanthamoeba castellanii* Neff, while the murine macrophage cell line (J774. A1) was used as the cytotoxicity model. A descriptive analysis of the results was conducted, and we could observe that 90% of the present compounds exhibited an inhibition of the parasite above 80%, while only 18.75% of the compounds inhibited the parasite with a lethality towards murine macrophage lower than 20% (Figure 1). Based on the cytotoxicity results, the availability of the drug on the market and an extensive bibliographic research, two compounds were chosen for further experiments: Almitrine and Terconazole.

### 2.2. In Vitro Activity of the Most Active Drugs against Trophozoite and Cyst of Various Acanthamoeba Strains (µM)

**Figure 2:**
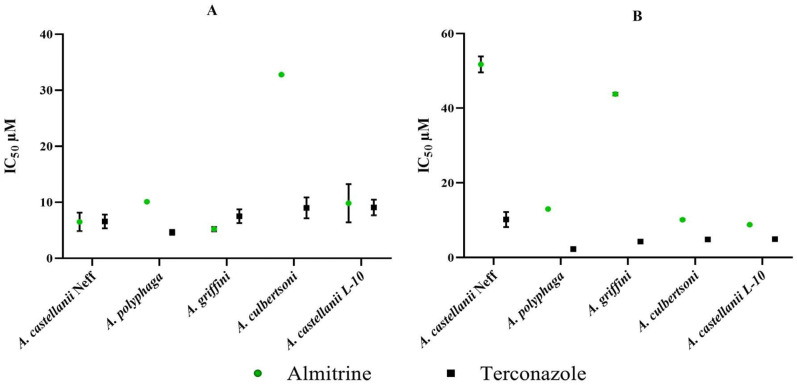
Distribution of IC_50_ of the trophocidal (**A**) and cysticidal (**B**) activities as a function of drugs and *Acanthamoeba* strains.

Both tested drugs, Almitrine and Terconazole, were able to inhibit the trophozoite and cyst stages of the five *Acanthamoeba* strains. The corresponding IC_50_ values are summarized in Figure 2. Parasites were separately incubated with six different concentrations at 26 °C. In the evaluation of the results, it could be observed that the amoebicidal activity was based on a dose-dependent application. The analysis of variance by two-way ANOVA illustrated that the amoebicidal activity was strain- and drug-dependent with *p* < 0.001. Among the strains tested, *Acanthamoeba castellanii* Neff was among the more sensitive in its trophozoite form, while its cyst form was among the more resistant, particularly to Almitrine. For the trophocidal effect, the IC_50_ of Almitrine ranged from 5.20 ± 0.4 µM to 32.08 ± 0.06 µM for *A. griffini* and *Acanthamoeba culbertsoni*, respectively, while the IC_50_ of Terconazole against *A. polyphaga* and *A. castellanii L10* was, respectively, 4.62 and 9.07 µM. As for the cysticidal effect, Almitrine had an IC_50_ ranging from 8.76 ± 0.07 to 51.72 ± 2.13 for *A. castellanii* L10 and *A. castellanii* Neff, respectively. According to the same figure and based on the ANOVA analysis, Terconazole was generally the more potent drug against both trophozoite and cyst forms with an IC_50_ towards cyst stage range of 2.24 ± 0.50 µM for *A. polyphaga* and 10.17 ± 2.01 µM for *A. castellanii* Neff (Supplementary File S1).

### 2.3. Characterization of the Type of Cell Death Induced by Terconazole on Acanthamoeba culbertsoni

Based on the amoebicidal activity, Terconazole was chosen to study the cell death induced in *Acanthamoeba culbertsoni* treated with a concentration corresponding to IC_90_. For each experiment, representative cell images were obtained by the confocal microscopy.

### 2.4. Disruption of the Cytoskeleton Organization in Acanthamoeba culbertsoni by the Terconazole 

In the present work, the effect of Terconazole on the two main cytoskeleton proteins, actin and tubulin, was investigated using a confocal microscope. 

### 2.5. Disruption of Actin Protein in Acanthamoeba culbertsoni

**Figure 3:**
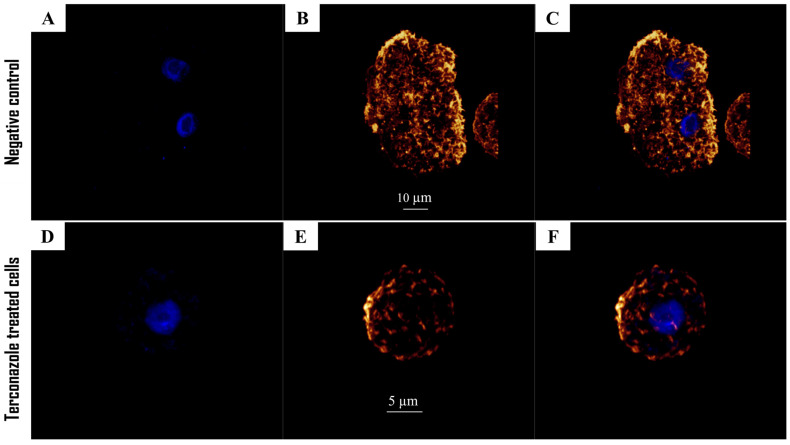
Evaluation of the effect of IC_90_ of Terconazole on actin distribution of the trophozoites of *Acanthamoeba culbertsoni*; cells show an undeveloped network with a dramatical decrease in cell size (**D**–**F**). The phalloidin-TRITC dye stains the polymerized actin cytoskeleton showing the normal organization of the networks with an orange fluorescence in the negative control cells (**A**–**C**). Mounting DAPI solution for DNA staining shows a blue fluorescence (**A**,**D**); Phalloidin-TRITC channel (**B**,**D**); overlay channel (**C**,**F**). All images (63×) were obtained using a Leica DMI 4000 B inverted confocal light microscope.

In the present work, specific staining of actin protein using a phalloidin-TRITC dye was performed for untreated and Terconazole-treated cells. As displayed in Figure 3, cells treated with the present azole emit lower fluorescence than the untreated cells, with a rounded shape and disappearance of the pseudopodium. 

### 2.6. Disruption of the Microtubule Network in Acanthamoeba culbertsoni

**Figure 4:**
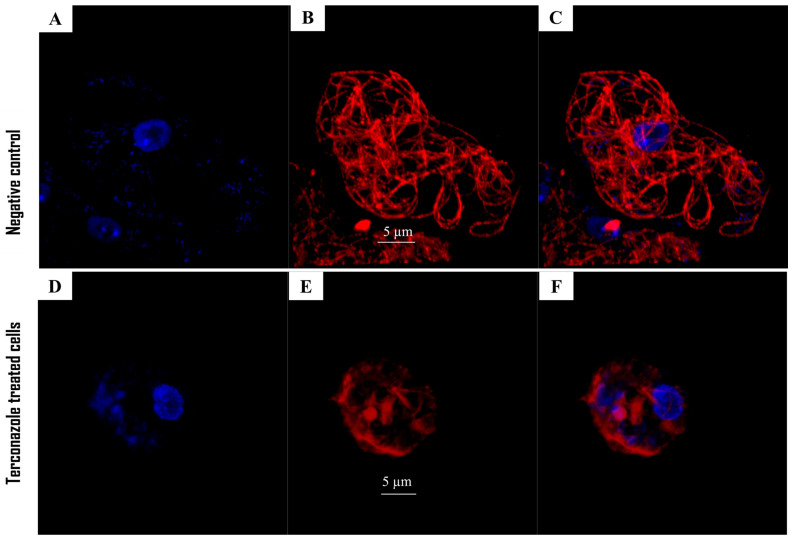
Trophozoites of *Acanthamoeba culbertsoni* incubated with IC_90_ of Terconazole for 24 h. Compared to the negative control (**A**–**C**), treated cells demonstrated an undeveloped tubulin network, with lower emitted red fluorescence (**D**–**F**). Mounting DAPI solution for DNA staining showed a blue fluorescence. Dapi channel (**A**,**D**); Alexa 594 channel (**B**,**E**); overlay channel (**C**,**F**) Images (63×) were obtained using a Leica DMI 4000 B inverted confocal light microscope.

In this study, an indirect immunofluorescence assay was performed to determine the effect of the present drug on the tubulin network of *Acanthamoeba* after 24 h of incubation, with IC_90_. We can assume the same effect as on actin filaments. In fact, a highly reduced cell shape with lower fluorescence could be observed (Figure 4), indicating the presence of uniformly distributed tubulin. 

### 2.7. Induced Chromatin Condensation in Acanthamoeba culbertsoni

**Figure 5:**
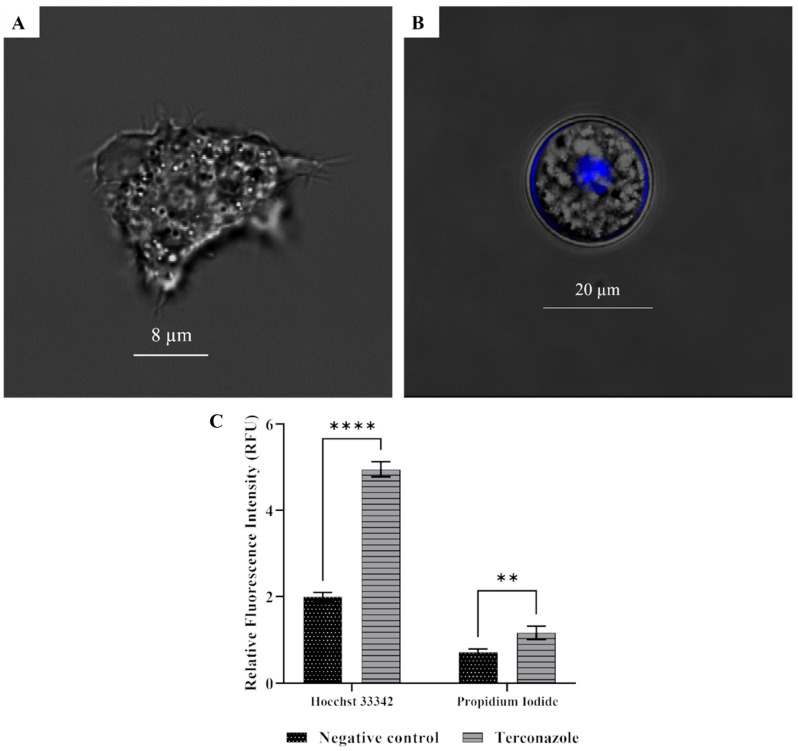
The effect of Terconazole at IC_90_ after 24 h of incubation on the chromatin condensation was conducted. Hoechst dye stains the condensate nucleic acids (**B**) with bright blue color in early apoptotic cells, while no chromatin changes are observed in negative control cells (**A**). EVOS M5000 and an objective of 40 were used to quantify the mean fluorescence intensity emitted by the cells stained. Differences between the values were assessed using one-way analysis of variance (ANOVA). When comparing to the negative control the result showed significant differences with ** *p* < 0.01 and  **** *p* < 0.0001 (**C**).

The effect of the present azole on the chromatin structure was studied using fluorescence microscopy with Hoechst 33324/PI staining. As shown in Figure 5, in treated cells we notice the presence of intensely bright blue, fluorescent cells. Some of the cells also emitted red fluorescence, implying that the cells were undergoing morphological changes compatible with an apoptosis-like pathway. 

### 2.8. Damaged Cell Plasma Membrane Permeability in Acanthamoeba culbertsoni

**Figure 6:**
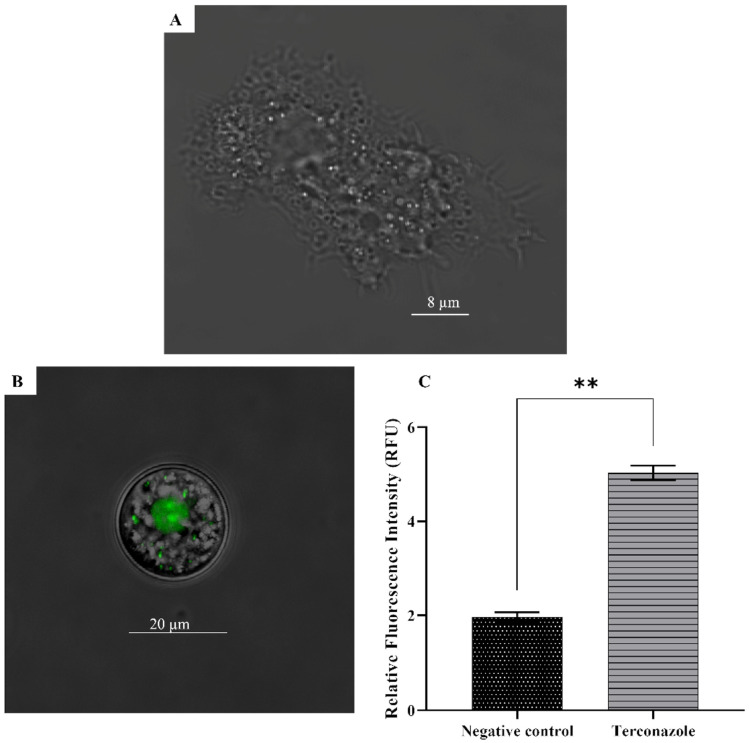
Upon binding to DNA, the intensity of SYTOX Green is enhanced 500 times, which is only possible when the plasma membrane is damaged and permeable to the dye (**B**). Negative control (**A**). The relative mean fluorescence was determined using the EVOS M5000 software and a one-way analysis of variance (ANOVA) was conducted. Data are presented as means ± SD, ** *p* < 0.01; the result shows significant differences when comparing treated cells with the negative control (**C**).

A cell-impermeable and highly DNA-binding dye, SYTOX green, was used to illustrate the eventual damage of Terconazole on cell membrane permeability. Although the treated cells emitted statistically higher green fluorescence than the negative control, their integrity was well maintained, as can be observed in Figure 6. 

### 2.9. Validation of the Mitochondrial Malfunction after Incubation with the Terconazole

In the present work, we studied the mitochondrial alterations by measuring the mitochondrial membrane potential, the ATP levels, and ROS production. 

### 2.10. Induction of a Collapse in the Mitochondrial Membrane Potential (MMP)

**Figure 7:**
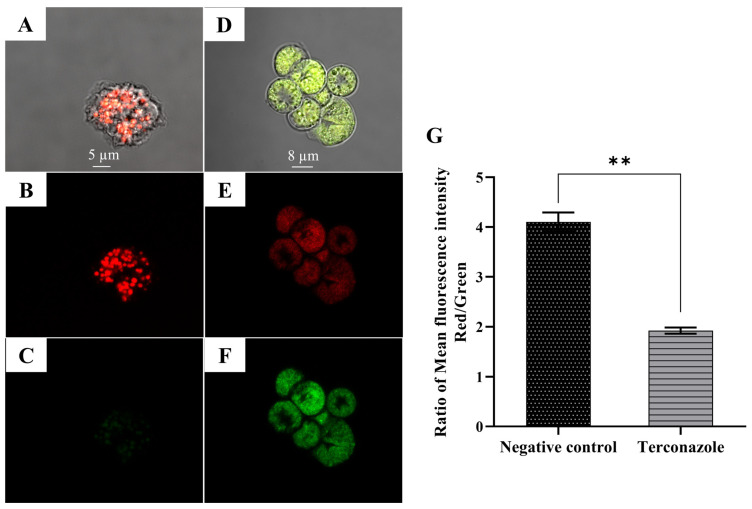
Evaluation of the mitochondrial membrane potential using JC-1 dye in *Acanthamoeba culbertsoni* trophozoites incubated with IC_90_ of Terconazole (**D**–**F**) for 24 h. Control cells (**A**–**C**). Images (63×) are based on Leica SPE confocal microscopy. Data showed in the graph are presented as means ± SD, ** *p* < 0.01; the results demonstrate significant differences when comparing cells treated with Terconazole to negative control cells (**G**). Differences between the mean values of ratio fluorescence intensity red/green were assessed using one-way analysis of variance (ANOVA). The mean fluorescence intensity of stained cells for each assay was determined using EVOS M5000. All experiments were conducted in triplicate.

The JC-1 dye was used to investigate the effect of the present drug on the mitochondrial membrane potential. This potential-dependent dye aggregates in the mitochondria with high MMP of healthy cells, while it diffuses across mitochondria to form a monomeric state upon depolarization in unhealthy cells [14]. The fluorescent dye accumulation in the mitochondria was optically detected by image-based fluorescence and confocal microscopies. Figure 7 demonstrates the aggregation of JC-1 within the negative control mitochondria, while in the treated cells we observed bright green fluorescence, indicating the presence of JC-1 monomers and a depolarization of the MMP. Using the EVOS M5000 software, a quantification of the mean fluorescence intensities for monomer (green) and red (aggregate) was conducted and the ratio of red fluorescence/green fluorescence was calculated. A 50% decrease in the MMP of the treated *Acanthamoeba* compared to the untreated cells can be observed (Figure 7G).

### 2.11. Decrease in the ATP Production

Constant mitochondrial malfunction typically results in a perturbation of the energy metabolism, leading to a decrease in the intracellular ATP levels. Accordingly, we proceeded to measure the total ATP levels produced after 24 h of treatment and relative to the negative control. We found that Terconazole produced a noticeable inhibition in the total ATP level by 62.5 ± 2.36%.

### 2.12. Increase in the Reactive Oxygen Species Production

**Figure 8:**
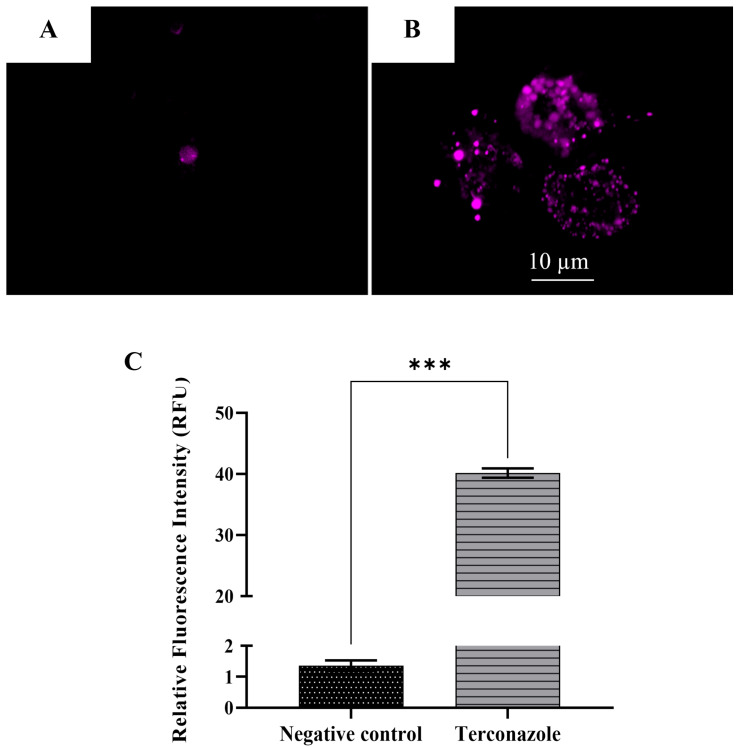
Evaluation of reactive oxygen species (ROS) production using a CellROX^®^ Deep Red fluorescent probe in *Acanthamoeba culbertsoni* trophozoites, incubated with IC_90_ of Terconazole (**B**) for 24 h. Control cells (**A**). The images (63×) were obtained using Leica SPE confocal microscopy. Data shown in the graph are presented as means ± SD, *** *p* < 0.001, obtained with the software of the EVOS™ FL Cell Imaging System M5000; the results demonstrate significant differences when comparing cells treated with nitroxoline to negative control (**C**). Differences between the values were assessed using one-way analysis of variance (ANOVA).

The overproduction of ROS was visualized using confocal and fluorescence microscopy using the CellROX^®^ deep red kit, a free radical specific dye. Healthy cells with reduced state emit weak fluorescence, while cells suffering from oxidative stress emit bright fluorescence. *Acanthamoeba* treated with Terconazole emitted significantly higher fluorescence than the untreated cells, demonstrating the increase in in situ ROS levels (Figure 8).

### 2.13. Highlighting Terconazole Effect on the Proteomic Profile of Acanthamoeba castellanii L10

**Figure 9:**
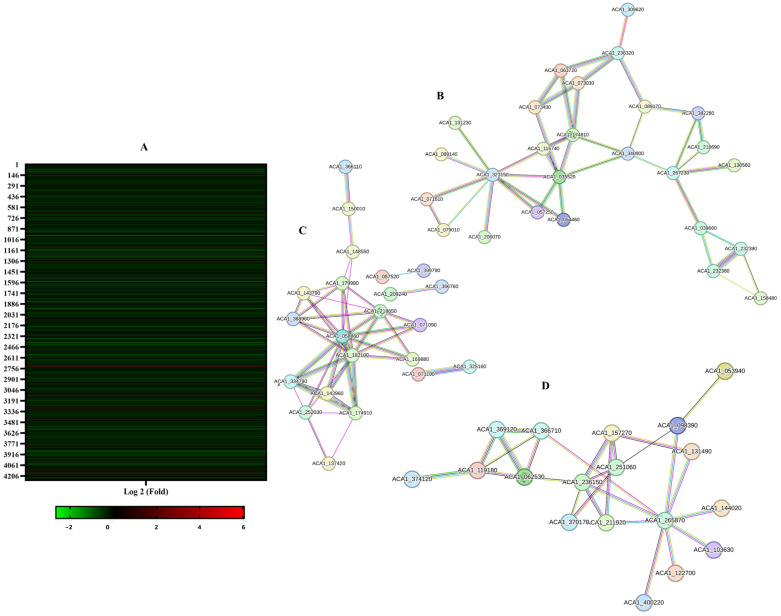
(**A**) Heatmaps of protein expression in treated cells relative to the negative control. Shown in these heatmaps are proteins that are significantly differentially expressed between treated and untreated cells. Downregulated proteins are represented in green color, while the upregulated proteins are in red. Representation of all down- and upregulated proteins with a median log 2 (FC). (**B**–**D**), cluster of protein–protein correlations, representing the elucidation of relationships between drug response pathways. Representation of protein–protein correlations obtained by the STRING free software. (**B**): Cluster of correlated proteins involved in sterol biosynthesis. (**C**): Cluster of correlated proteins involved in translation and RNA ribosomal proteins. (**D**): Cluster of correlated proteins involved in DNA replication including DNA polymerase.

To further understand the mode of action of Terconazole on *Acanthamoeba*, a proteomic profiling of both untreated and treated cells via mass spectrometry-based proteomic approach was conducted. To do so, two samples of the trophozoite stage of *Acanthamoeba castellanii* L10 were prepared: untreated and Terconazole treated cells with IC_50_ of Terconazole. After incubation for 24 h, proteins were prepared as mentioned in the Material and Methods section. Proteomic analysis led to the identification of 4566 proteins. Based on the statistical analysis and compared to the negative control, a total of 223 proteins were at least twofold differentially expressed after Terconazole treatment. Among these proteins, as can be observed in the heatmap in Figure 9A, most of the affected proteins were downregulated with a percentage of 80%, whereas Terconazole activated 20% of the affected proteins.

To better understand the drug pathway and the presence of eventual relationships among the downregulated proteins, we used the STRING database v 12.0 to present the protein–protein relationships. Three clusters of protein–protein correlations were found, as illustrated in Figure 9B–D. Most of the affected proteins were involved in DNA replication, RNA translation, and metabolic steroid biosynthesis. 

## 3. Discussion 

Recently, due to the COVID-19 epidemic, drug repurposing has gained great attention. Drug repositioning, as well as repurposing, refers to the use of available drugs for a different disease [15]. This strategy has the benefit of skipping various phases that are essential in the validation of a new drug, including efficacy, dose, formulation, and eventual toxicity [16]. In infectious diseases, drug redirection can overcome microbial resistance. In the present work, the COVID box was first evaluated against a type strain of *Acanthamoeba*: *Acanthamoeba castellanii* Neff. Almitrine and Terconazole are among the most active drugs with low cytotoxicity that can be purchased in the market. Although Almitrine presents lower cytotoxicity than Terconazole, it was discarded due to its low solubility. Terconazole is the latest class of azoles belonging to the triazoles family, which are characterized by better solubility, which in turn increases their bioavailability and hence their activity. They were also reported to have greater selectivity index towards the pathogens [17]. In *Acanthamoeba*, azoles have been reported to be active throughout the inhibition of ergosterol production [18]. Shing et al. (2021) reviewed the azole activity in the present parasite and reported that azoles including posaconazole, isavuconazole, clotrimazole, voriconazole, itraconazole, and miconazole have a strong trophocidal effect, while posaconazole and isavuconazole have been reported to also be cysticidal agents [19]. Terconazole, known to be one of the most effective triazoles against *Candida* species, has been reported to exhibit further biological activities (including *Trypanosoma cruzi*) and to enhance the activity of antimitotic drugs by inducing apoptosis and G2 arrest in cancer cells [20,21,22]. In a previous work, we reported the activity of Terconazole against *Naegleria fowleri* with an IC_50_ of 3.68 ± 0.61 µM [13]. Despite all the articles that have reported the antifungal activity of various azoles, only a few have shown the mechanism of cell death. Herein, we demonstrated using fluorescence microscopy that Terconazole can trigger various cellular events that conform to the programmed cell death, such as chromatin condensation and mitochondrial malfunction. In this regard, Shuzhen et al. (2022) demonstrated that bifonazole can induce cell death in *Penicillium expansum* via cell membrane damage and mitochondrial malfunction resulting in the accumulation of ROS [23]. The authors reported that the initiation of apoptosis-like death in the fungus could be related to ergosterol depletion. 

To deepen our understanding of how Terconazole could trigger apoptosis-like death in *Acanthamoeba*, a proteomic profile analysis was conducted for untreated and IC_50_-treated *Acanthamoeba castellanii* L10. Among the most downregulated proteins, three protein–protein clusters could be observed. The most important cluster was specifically enrolled in metabolic and sterol biosynthesis pathways. Among the inhibited proteins, we can highlight the protein ACA1_174810 corresponding to the Cytochrome p450 superfamily protein. This protein has been cited as the principal target of the azole in fungus, yeast, and *Acanthamoeba* treatment [24,25,26]. The inhibition of the sterol biosynthesis, namely ergosterol in *Acanthamoeba*, can lead to cell membrane damage. In the same cluster, various oxidoreductase proteins, including the NAD-specific glutamate dehydrogenase and sulfite reductase (NADPH), were also inhibited by Terconazole, confirming the conclusion of She et al. (2020), who reported the relationship between the ergosterol biosynthesis and the cAMP pathway and NADH oxidation in the mitochondria [27]. 

The second cluster involved the inhibition of the translation pathway by inhibiting RNA ribosomal proteins such as ACA1_218650 and ACA1_143790. Recently, targeting the depletion of mRNA translation has constituted a promising approach in cancer therapy [28]. Among the inhibited RNA proteins, we disclose the repression of the mitochondrial ribosomal protein (ACA1_179980) and the DEAD box RNA helicase (ACA1_157270). Mitochondrial ribosomal proteins play a crucial role in the formation of the mitochondrial ribosome and are involved in the oxidative phosphorylation protein synthesis. Depletion of the mitoribosome can result in the inhibition of the oxidative phosphorylation pathways and the proper functioning of the mitochondria [29]. The DEAD box RNA helicases constitute a large family of proteins involved in various aspects of RNA metabolism including translation and ribosome biogenesis [30]. The downregulation of this ATP-dependent protein could be the result of the decrease in the intracellular ATP levels. Lu Zhang and Xiaogang Li (2021) reported that the knockdown of different RNA helicases can lead to cell cycle arrest and the activation of the cell death program [31]. The third protein–protein cluster obtained involves the downregulation of proteins implicated in DNA binding and replication. Among these proteins, we cite the DNA topoisomerase 2, suppression of which can trigger cell death by the apoptosis pathway [32]. A second protein to highlight is the DNA polymerase, whose main role consists of DNA replication, as well as DNA repair pathways [33]. Inhibition of this enzyme could be fatal to the cells and is a considered an important target for the treatment of numerous hyperproliferative diseases like viral infections, autoimmune disorders, and cancer [34].

## 4. Conclusions

Taken together, among the 160 compounds tested from the COVID Box, Terconazole was selected as a promising amoebicidal drug against *Acanthamoeba castellanii* Neff. Using fluorescence staining and proteomic analysis, we can suggest that Terconazole inhibits *Acanthamoeba culbertsoni and Acanthamoeba castellanii* L10 through the inhibition of ergosterol synthesis and induction of PCD via ROS production. However, studies including the evaluation of the effect on *Acanthamoeba* in ex vivo and in vivo model should be conducted to validate its efficacy.

## 5. Materials and Methods

### 5.1. Drugs and Chemicals

The COVID Box was kindly donated by the Medicines for Malaria Venture (MMV) foundation. All compounds were dissolved in DMSO at 10 mM.

### 5.2. Acanthamoeba Strains

The initial trophocidal activity of the COVID Box compounds was studied against the ATCC type strain *Acanthamoeba castellanii* Neff genotype T4 (ATCC 30010). Further strains were used to fine-tune the most active amoebicidal agents: *A. polyphaga* (genotype T4, type strain: ATCC 30461), *A. griffini* (genotype T3, clinical strain), *A. castellanii* L-10 (genotype T4, clinical strain), and *A. culbertsoni* (genotype T10, type strain: ATCC 30171). For the PCD and proteomic studies, *Acanthamoeba culbertsoni* and *Acanthamoeba castellanii* L10, respectively, were used. All strains were grown axenically in Peptone Yeast Glucose (PYG) medium (0.75% (*w*/*v*) proteose peptone, 0.75% (*w*/*v*) yeast extract, and 1.5% (*w*/*v*) glucose) containing 40 µg of gentamicin mL^−1^ (Biowest, Nuaillé, France). 

### 5.3. In Vitro Effect against the Trophozoite Stage of Acanthamoeba

The trophocidal effect of the 160 molecules from the COVID Box was determined using the alamarBlue™ (Life Technologies, Madrid, Spain) assay as previously described with modifications [35,36,37]. Briefly, *Acanthamoeba castellanii* Neff trophozoites were first seeded in triplicate on a 96-well microtiter plate with 99 μL from a stock solution of 5 × 10^4^ cells mL^−1^. Subsequently, 1 μL of each compound was added to each well to assess the in vitro amoebicidal activity at 10 μM as the final concentration. Finally, 10 µL of alamarBlue™ Cell Viability Reagent (Bioresource, Europe, Nivelles, Belgium) was added into each well. The plates were then incubated for 96 h at 28 °C with a slight agitation. An EnSpire microplate reader (PerkinElmer, Waltham, MA, USA) was used to measure fluorescence at 570/585 nm. Then, the percentage of inhibition value of the tested COVID Box compounds was determined in comparison with the negative control.

Almitrine and Terconazole were the COVID Box products selected to evaluate the amoebicidal effect against the trophozoite stage of six strains of *Acanthamoeba: A. castellanii* Neff, *A. griffini, A. polyphaga, A. culbertsoni*, and *A. castellanii* L10. The inhibition concentration 50 (IC_50_) value was calculated based on the alamarBlue™ assay, as previously mentioned.

### 5.4. In Vitro Effect against the Cysts Stage

Based on the amoebicidal and cytotoxicity results, the selected compounds, Almitrine and Terconazole, were further investigated on the cyst stage of the various *Acanthamoeba* strains: *A. castellanii* Neff, *A. griffini, A. polyphaga, A. culbertsoni*, and *A. castellanii* L-10. The cysticidal activity was assessed by the alamarBlue™ method, as described previously [38]. Briefly, in a 96-well plate, a serial dilution of the mentioned drugs was first conducted. Later, cysts were resuspended in PYG medium and were added into each well at a concentration of 5 × 10^4^ cysts mL^−1^. The plates were examined over a week using an inverted microscope. After 7 days of incubation, the plates were centrifuged at 3000 rpm and the medium was replaced with a new PYG. Finally, 10 μL of alamarBlue^TM^ Cell Viability Reagent (Biosource, Europe, Nivelles, Belgium) was placed into each well, and the plates were incubated at 28 °C with slight agitation. The emitted fluorescence was measured after 144 h.

### 5.5. Evaluation of the Cytotoxicity against Murine Macrophage

The cytotoxicity of the COVID Box compounds was evaluated against murine macrophages of the J774A.1 cell line (ATCC TIB-67), as previously described [36]. As mentioned in the trophocidal assay, cells were allowed to adhere on a 96-well plate in 37 °C in a 5% CO_2_ humidified incubator. Later, a well-defined volume of the compounds was added at an affinal concentration of 10 µM to each well. Fluorescence emitted by the alamarBlue^TM^ was measured after 24 h of incubation and the percentage inhibition value was determined. The Cytotoxicity Concentration 50 (CC_50_) value of Almitrine and Terconazole was determined following the same method described previously for the trophocidal effect. 

### 5.6. Fluorescence Microscopy 

#### 5.6.1. Immunofluorescence Staining of Tubulin

Immunofluorescence staining of tubulin was performed according to the manufacturer’s staining procedure (Sigma-Aldrich, Madrid, Spain) with slight modifications. Briefly, cells were treated first with Terconazole at IC_90_. After 24 h of incubation, 50 µL of treated and untreated cell suspensions was placed on a gelatin precoated coverslip for 30 min. Cells were first fixed with formaldehyde (4%) and later permeabilized with Triton (0.3%) for 10 min. Washed with PBS (3X), cells were treated with 5% BSA in PBS 1X/150 mM sucrose for 30 min and washed with glycine 100 mM. At this stage, the trophozoites were incubated with a monoclonal antibody, Anti-α-Tubulin (1:2000), for 2 h at room temperature. Washed twice with PBS, cells were then incubated with the second antibody labeled with Alexa 594 (1:500; from Thermo Fisher Scientific, Rockford, IL, USA) for an hour at room temperature in darkness. Finally, cells were washed with PBS 1X and mounted using mounting medium with DAPI (Sigma-Aldrich; Madrid, Spain). Three-dimensional and maximum projection imaging of the trophozoites was performed by Z-stack imaging using a Leica DMI 4000 B inverted confocal microscope with LAS X software (Leica DMI 4000 B inverted confocal microscope), a 405 nm laser and a 532 nm laser, and a Leica HCX PL Apo 63× Oil Objective.

#### 5.6.2. Acting Staining Using Phalloidin-TRITC Conjugate

As in the immunofluorescence staining, cells were first treated with IC_90_ of Terconazole and fixed with formaldehyde (4%) over a gelatin-precoated coverslip. Later, the cells were permeabilized with Triton (0.1%), followed by a 1 h incubation with phalloidin–tetramethylrhodamine B isothiocyanate (phalloidin-TRITC; Sigma-Aldrich, Madrid, Spain) at room temperature. Cells were finally washed with PBS 1X and mounted using mounting medium with DAPI. Cells were examined by Z-stack imaging using a Leica DMI 4000 B inverted confocal microscope with a 63× objective (Leica Microsystems, Wetzlar, Germany), a 405 nm laser, and a 532 nm laser. 

### 5.7. Mode of Action Evaluation

*Acanthamoeba culbertsoni* cells were incubated with IC_90_ of Terconazole for 24 h. All mean fluorescence intensities were quantified using EVOS M5000 (Life Technologies, Madrid, Spain), processing three different observations in triplicate using an objective (×40), and images of single cells were obtained by a Leica DMI 4000 B inverted confocal microscope with a 63× objective (Leica Microsystems, Germany). All assays were performed in triplicate.

#### 5.7.1. Double-Stain Assay for Programmed Cell Death Determination

A double-stain apoptosis detection kit (Chromatin Condensation/Dead Cell Apoptosis Kit) with Hoechst 33342 and Propidium Iodide (Invitrogen, Madrid, Spain) were used. The experiment was carried out by following the manufacturer’s recommendations [38].

#### 5.7.2. Plasma Membrane Permeability

The effect of the study drug on the cell’s membrane permeability was assessed using specific DNA staining. SYTOX^®^ Green (Molecular Probes, Invitrogen, Madrid, Spain) is a DNA-specific dye impermeable to the cells, whose fluorescence increases 500× upon fixation to the DNA. Briefly, 105 trophozoites were incubated for 24 h with IC_90_ of the drug solution. Subsequently, the SYTOX^®^ Green dye was added at a final concentration of 1 μM for 15 min in the dark [38]. 

#### 5.7.3. Analysis of Mitochondrial Membrane Potential Using JC-1 Kit

Based on the JC-1 state and the emitted fluorescence, we distinguished two sets in the cellular population: live cells emit high red fluorescence and affected cells with collapsed mitochondrial membrane potential emit a higher green fluorescence [38]. 

#### 5.7.4. ATP Assays

After 24 h of drug treatment, the intracellular ATP content was measured using a CellTiter-Glo Luminescent Cell Viability Assay (Promega, Madison, WI, USA). The experiment was carried out according to the manufacturer’s instructions, and the luminescence intensity was measured in a white 96-well plate using an EnSpire^®^ Multimode Plate Reader. 

#### 5.7.5. Estimation of ROS Generation Using CellROX^®^ Deep Red Staining

The overproduction of intracellular ROS was measured using the CellROX^®^ Deep red fluorescent probe (Invitrogen, Madrid, Spain). 

#### 5.7.6. Comparative Label-Free Proteomics

The proteomic analysis was conducted according to Arbon et al., 2022 [39]. A quantity of 10^6^ cells was first incubated with Terconazole at IC_50_ for 24 h, then treated and untreated cells were centrifuged and washed with PBS. Both groups were prepared in three biological replicates. The assay was conducted as detailed in our previous work [38]. Results were analyzed using STRING free software and GraphPad 9 prism (Supplementary Material File S2). 

### 5.8. Statistical Analysis

All data are expressed as the mean ± standard deviation of at least three independent experiments. To highlight the effects of drug and tested strains, a statistical comparison was conducted using two-way ANOVA. All analyses and graphics were conducted with GraphPad Prism version 9.0 (GraphPad Software, San Diego, CA, USA). Statistical significance was set at *p* < 0.05.

## Data Availability

The original contributions presented in the study are included in the article/supplementary material, further inquiries can be directed to the corresponding authors.

## Supplementary Materials

The following supporting information can be downloaded at: https://www.mdpi.com/article/10.3390/ph17060808/s1, File S1: Amoebicidal activity (IC_50_) of almitrine and terconazole against trophozoite and cyst stages of *Acanthamoeba* spp. File S2: Experimental conditions for the proteomic analysis. References [40,41,42,43] are cited in the Supplementary Materials (S2).

## References

[1] Reyes-Batlle M., Córdoba-Lanús E., Domínguez-de-Barros A., Sifaoui I., Rodríguez-Expósito R.L., Mantesa-Rodríguez S., Piñero J.E., Lorenzo-Morales J. (2024). Reliable and specific detection of *Acanthamoeba* spp. in dishcloths using quantitative real-time PCR assay. Food Microbiol..

[2] Siddiqui R., Khan N.A. (2012). Biology and pathogenesis of *Acanthamoeba*. Parasites Vectors.

[3] Wang Y., Jiang L., Zhao Y., Ju X., Wang L., Jin L., Fine R.D., Li M. (2023). Biological characteristics and pathogenicity of Acanthamoeba. Front. Microbiol..

[4] Fanselow N., Sirajuddin N., Yin X., Huang A.J.W., Stuart P.M. (2021). Acanthamoeba Keratitis, Pathology, Diagnosis and Treatment. Pathogens.

[5] Sifaoui I., Reyes-Batlle M., López-Arencibia A., Chiboub O., Bethencourt-Estrella C.J., San Nicolás-Hernández D., Rodríguez Expósito R.L., Rizo-Liendo A., Piñero J.E., Lorenzo-Morales J. (2019). Screening of the pathogen box for the identification of anti-Acanthamoeba agents. Exp. Parasitol..

[6] Botella L.M. (2022). Drug repurposing as a current strategy in medicine discovery. Semer. Med. Fam..

[7] Barnes C.B.G., Dans M.G., Jonsdottir T.K., Crabb B.S., Gilson P.R. (2022). PfATP4 inhibitors in the Medicines for Malaria Venture Malaria Box and Pathogen Box block the schizont-to-ring transition by inhibiting egress rather than invasion. Front. Cell. Infect. Microbiol..

[8] Samby K., Willis P.A., Burrows J.N., Laleu B., Webborn P.J.H. (2021). Actives from MMV Open Access Boxes? A suggested way forward. PLoS Pathog..

[9] Lopez-Arencibia A., Sifaoui I., Reyes-Batlle M., Bethencourt-Estrella C.J., San Nicolas-Hernandez D., Lorenzo-Morales J., Pinero J.E. (2021). Discovery of New Chemical Tools against Leishmania amazonensis via the MMV Pathogen Box. Pharmaceuticals.

[10] Duffy S., Sykes M.L., Jones A.J., Shelper T.B., Simpson M., Lang R., Poulsen S., Sleebs B.E., Avery V.M. (2017). Screening the Medicines for Malaria Venture Pathogen Box across Multiple Pathogens Reclassifies Starting Points for Open-Source Drug Discovery. Antimicrob. Agents Chemother..

[11] Spalenka J., Escotte-Binet S., Bakiri A., Hubert J., Renault J., Velard F., Duchateau S., Aubert D., Huguenin A., Villena I. (2018). Discovery of New Inhibitors of Toxoplasma gondii via the Pathogen Box. Antimicrob. Agents Chemother..

[12] Dos Santos B.R., Ramos A.B.d.S.B., de Menezes R.P.B., Scotti M.T., Colombo F.A., Marques M.J., Reimao J.Q. (2023). Repurposing the Medicines for Malaria Venture’s COVID Box to discover potent inhibitors of Toxoplasma gondii, and in vivo efficacy evaluation of almitrine bismesylate (MMV1804175) in chronically infected mice. PLoS ONE.

[13] Chao-Pellicer J., Arberas-Jiménez I., Sifaoui I., Piñero J.E., Lorenzo-Morales J. (2024). Exploring therapeutic approaches against Naegleria fowleri infections through the COVID box. Int. J. Parasitol. Drugs Drug Resist..

[14] Sivandzade F., Bhalerao A., Cucullo L. (2019). Analysis of the Mitochondrial Membrane Potential Using the Cationic JC-1 Dye as a Sensitive Fluorescent Probe. Bio Protoc..

[15] Hua Y., Dai X., Xu Y., Xing G., Liu H., Lu T., Chen Y., Zhang Y. (2022). Drug repositioning: Progress and challenges in drug discovery for various diseases. Eur. J. Med. Chem..

[16] Pareek S., Huang Y., Nath A., Huang R.S., To K.K.W., Cho W.C.S. (2020). Chapter 6—The success story of drug repurposing in breast cancer. Drug Repurposing in Cancer Therapy.

[17] Teixeira M.M., Carvalho D.T., Sousa E., Pinto E. (2022). New Antifungal Agents with Azole Moieties. Pharmaceuticals.

[18] Henriquez F.L., Ingram P.R., Muench S.P., Rice D.W., Roberts C.W. (2007). Molecular Basis for Resistance of Acanthamoeba Tubulins to All Major Classes of Antitubulin Compounds. Antimicrob. Agents Chemother..

[19] Shing B., Balen M., Mckerrow J.H., Debnath A. (2022). Acanthamoeba Keratitis: An update on amebicidal and cysticidal drug screening methodologies and potential treatment with azole drugs. Expert Rev. Anti Infect. Ther..

[20] Bahy R., Helal D. (2022). Evaluation of the Antimycotic activity of Terconazole proniosomal Gel. Egypt. J. Med. Microbiol..

[21] Lee J.S., Oh Y., Park J.H., Kyung S.Y., Kim H.S., Yoon S. (2022). Terconazole, an Azole Antifungal Drug, Increases Cytotoxicity in Antimitotic Drug-Treated Resistant Cancer Cells with Substrate-Specific P-gp Inhibitory Activity. Int. J. Mol. Sci..

[22] Reigada C., Saye M., Valera-Vera E., Miranda M.R., Pereira C.A. (2019). Repurposing of terconazole as an anti Trypanosoma cruzi agent. Heliyon.

[23] Yang S., Yan D., Li M., Li D., Zhang S., Fan G., Peng L., Pan S. (2022). Ergosterol depletion under bifonazole treatment induces cell membrane damage and triggers a ROS-mediated mitochondrial apoptosis in Penicillium expansum. Fungal Biol..

[24] Pratiwi R.A., Yahya N.S.W., Chi Y. (2022). Bio function of Cytochrome P450 on fungus: A review. IOP Conf. Ser. Earth Environ. Sci..

[25] Yoshida Y. (1988). Cytochrome P450 of fungi: Primary target for azole antifungal agents. Curr. Top. Med. Mycol..

[26] Huang J., Ko P., Huang C., Wen P., Chen C., Shih M., Lin W., Huang F. (2021). Cytochrome P450 monooxygenase of Acanthamoeba castellanii participates in resistance to polyhexamethylene biguanide treatment. Parasite.

[27] She X., Zhang L., Peng J., Zhang J., Li H., Zhang P., Calderone R., Liu W., Li D. (2020). Mitochondrial Complex I Core Protein Regulates cAMP Signaling via Phosphodiesterase Pde2 and NAD Homeostasis in Candida albicans. Front. Microbiol..

[28] Taylor J., Yeomans A.M., Packham G. (2020). Targeted inhibition of mRNA translation initiation factors as a novel therapeutic strategy for mature B-cell neoplasms. Explor. Target. Anti-Tumor Ther..

[29] Mo D., Liu C., Chen Y., Cheng X., Shen J., Zhao L., Zhang J. (2023). The mitochondrial ribosomal protein mRpL4 regulates Notch signaling. EMBO Rep..

[30] Lindqvist L., Oberer M., Reibarkh M., Cencic R., Bordeleau M., Vogt E., Marintchev A., Tanaka J., Fagotto F., Altmann M. (2008). Selective pharmacological targeting of a DEAD box RNA helicase. PLoS ONE.

[31] Zhang L., Li X. (2021). DEAD-Box RNA Helicases in Cell Cycle Control and Clinical Therapy. Cells.

[32] Yildizhan H., Barkan N.P., Karahisar Turan S., Demiralp Ö., Özel Demiralp F.D., Uslu B., Ōzkan S.A., Grumezescu A.M. (2018). Chapter 1—Treatment strategies in cancer from past to present. Drug Targeting and Stimuli Sensitive Drug Delivery Systems.

[33] Garcia-Diaz M., Bebenek K. (2007). Multiple functions of DNA polymerases. CRC Crit. Rev. Plant Sci..

[34] Berdis A.J. (2017). Inhibiting DNA Polymerases as a Therapeutic Intervention against Cancer. Front. Mol. Biosci..

[35] Martín-Navarro C.M., Lorenzo-Morales J., Cabrera-Serra M.G., Rancel F., Coronado-Álvarez N.M., Piñero J.E., Valladares B. (2008). The potential pathogenicity of chlorhexidine-sensitive Acanthamoeba strains isolated from contact lens cases from asymptomatic individuals in Tenerife, Canary Islands, Spain. J. Med. Microbiol..

[36] Sifaoui I., Reyes-Batlle M., López-Arencibia A., Wagner C., Chiboub O., De Agustino Rodríguez J., Rocha-Cabrera P., Valladares B., Piñero J.E., Lorenzo-Morales J. (2017). Evaluation of the anti-Acanthamoeba activity of two commercial eye drops commonly used to lower eye pressure. Exp. Parasitol..

[37] Sifaoui I., Reyes-Batlle M., López-Arencibia A., Chiboub O., Rodríguez-Martín J., Rocha-Cabrera P., Valladares B., Piñero J.E., Lorenzo-Morales J. (2018). Toxic effects of selected proprietary dry eye drops on Acanthamoeba. Sci. Rep..

[38] Rodríguez-Expósito R.L., Sifaoui I., Reyes-Batlle M., Fuchs F., Scheid P.L., Piñero J.E., Sutak R., Lorenzo-Morales J. (2023). Induction of Programmed Cell Death in Acanthamoeba culbertsoni by the Repurposed Compound Nitroxoline. Antioxidants.

[39] Arbon D., Zeniskova K., Subrtova K., Mach J., Stursa J., Machado M., Zahedifard F., Lestinova T., Hierro-Yap C., Neuzil J. (2022). Repurposing of MitoTam: Novel Anti-Cancer Drug Candidate Exhibits Potent Activity against Major Protozoan and Fungal Pathogens. Antimicrob. Agents Chemother..

[40] Hughes C.S., Moggridge S., Müller T., Sorensen P.H., Morin G.B., Krijgsveld J. (2019). Single-pot, solid-phase-enhanced sample preparation for proteomics experiments. Nat. Protoc..

[41] Rappsilber J., Mann M., Ishihama Y. (2007). Protocol for micro-purification, enrichment, pre-fractionation and storage of peptides for proteomics using StageTips. Nat. Protoc..

[42] Cox J., Hein M.Y., Luber C.A., Paron I., Nagaraj N., Mann M. (2014). Accurate proteome-wide label-free quantification by delayed normalization and maximal peptide ratio extraction, termed MaxLFQ. Mol. Cell Proteomics.

[43] Tyanova S., Temu T., Sinitcyn P., Carlson A., Hein M.Y., Geiger T., Mann M., Cox J. (2016). The Perseus computational platform for comprehensive analysis of (prote)omics data. Nat. Methods.

