# Supplementary material for: Amoebicidal Effect of COVID Box Molecules against Acanthamoeba: A Study of Cell Death
Source: Pharmaceuticals (Basel). 2024 Jun 20;17(6):808. doi: 10.3390/ph17060808 (PMC11206913; doi:10.3390/ph17060808)
Supplement: Supplementary file 1 [file pharmaceuticals-17-00808-s001.zip › pharmaceuticals-3039689-Supplementary S1.pdf]

Table 1: Amoebicidal activity (IC_{50}) of Almitrine against both trophozoite and cyst stages of five strains of *Acanthamoeba* , along with the assessment of the cytotoxic concentration (CC_{50}) in murine macrophages and their selectivity index (SI). All data were obtained from the mean of the 3 replicates of each assay, with their corresponding standard deviation.

	***A. castellanii* Neff**		***A. polyphaga***		***A. griffini***		***A. culberstoni***		***A. castellanii* L10**	
	Trophozoite stage	Cyst stage	Trophozoite stage	Cyst stage	Trophozoite stage	Cyst stage	Trophozoite stage	Cyst stage	Trophozoite stage	Cyst stage
IC_{50} µM	6,50 ± 1.65	51,72 ± 2.13	10,09 ± 0.27	12,96 ± 0.16	5,20 ± 0.40	43,78 ± 0.35	32,79 ± 3.05	10,11 ± 0.18	9,82 ± 10.18	8,76 ± 0.07
CC_{50} µM	> 100									
SI (CC_{50}/ IC_{50})	> 15,38	> 1,93	> 9,91	>7,72	>19,23	>2,28	>3,05	>9,89	>10,18	>11,42

Table 2: Amoebicidal activity (IC_{50}) of Terconazole against both trophozoite and cyst stages of five strains of *Acanthamoeba*, along with the assessment of the cytotoxic concentration (CC_{50}) in murine macrophages and their selectivity index (SI). All data were obtained from the mean of the 3 replicates of each assay, with their corresponding standard deviation.

	***A. castellanii* Neff**		***A. polyphaga***		***A. griffini***		***A. culberstoni***		***A. castellanii* L10**	
	Trophozoite stage	Cyst stage	Trophozoite stage	Cyst stage	Trophozoite stage	Cyst stage	Trophozoite stage	Cyst stage	Trophozoite stage	Cyst stage
IC_{50} µM	6,57 ± 1,73	10,17 ± 2,01	4,62 ± 0,61	2,22 ± 0,51	7,50 ± 1,73	4,24 ± 19,95	9,00 ± 2,63	4,80 ± 0,35	9,07 ± 1,97	4,88 ± 0,01
CC_{50} µM	84.6 ± 15.58									
SI (CC_{50}/ IC_{50})	12,88	8,32	18,31	38,11	11,27	19,95	9,40	17,63	9,33	17,32
