# Supplementary material for: Amoebicidal Effect of COVID Box Molecules against Acanthamoeba: A Study of Cell Death
Source: Pharmaceuticals (Basel). 2024 Jun 20;17(6):808. doi: 10.3390/ph17060808 (PMC11206913; doi:10.3390/ph17060808)
Supplement: Supplementary file 1 [file pharmaceuticals-17-00808-s001.zip › pharmaceuticals-3039689-Supplementary S2.pdf]

Table 1 [40-43]

**Sample preparation**	Samples were homogenised and lysed by boiling at 95°C for 10 min in 100mM TEAB (Triethylammonium bicarbonate) containing 2% SDC (sodium deoxycholate), 40mM chloroacetamide, 10mM TCEP (Tris(2-carboxyethyl)phosphine) and further sonicated (Bandelin Sonoplus Mini 20, MS 1.5). Protein concentration was determined using BCA protein assay kit (Thermo) and 30 µg of protein per sample was used for MS sample preparation. Samples were further processed using SP3 beads according to (1). Finally, samples were acidified with TFA to 1% final concentration and peptides were desalted using in-house made stage tips packed with C18 disks (Empore) according to (2)
**Enzyme specificity**	Trypsin/P with max 2 miscleavages and minimal and maximal peptide length 7 and 25 amino acids respectively
**Trap column**	C18 PepMap100, 5 µm particle size, 300 µm x 5 mm (Thermo Scientific) 4 min, 18 µl/min; loading buffer (2% acetonitrile, 0.1% trifluoroacetic acid, water); elution with mobile phase gradient from 4% to 35% in 120 min; 1 ug of sample was loaded on the column
**LC-MS column**	Nano Reversed phase column (EASY-Spray column, 50 cm x 75 µm ID, PepMap C18, 2 µm particles, 100 Å pore size)
**Mobile phases**	Mobile phase buffer A (water and 0.1% formic acid); mobile phase B (acetonitrile and 0.1% formic acid)
**Peptides analysis**	Gas-phase ions were analyzed by a Thermo Orbitrap Fusion (Q-OT- qIT, Thermo Scientific); survey scans from 350 to 1400 m/z in orbitrap; resolution - 120K (200 m/z); target ion count - 5×10^5; tandem MS - 1,5 Th quadrupol; HCD fragmentation collision energy - 30; rapid scan MS analysis in the ion trap; MS2 ion count target - 10^4; max injection time - 35 ms; precursors with charge state 2–6 were sampled for MS2; dynamic exclusion duration - 45 s with a 10 ppm tolerance; top speed mode; 2 s cycles
**Peak list, search engine**	All data were processed by MaxQuant 2.3.1.0 with built-in search engine Andromeda, which was also used for peak list generation, peptides assignment to proteins and data normalization (according to (3)); MS1 maximum peak intensity was used for quantification
**Sequence database**	AmoebaDB-65_AcastellaniiNeff_AnnotatedProteins database (downloaded from https://amoebadb.org/ at 1.6.2023, containing 14 979 entries)
**Data analysis**	Analysis was performed using Perseus 1.6.15.0 software (4); statistical relevance was estimated using students t-test with Benjamini-Hochberg's correction (FDR = 0.05)
**Fixed modifications**	Carbamidomethyl on cysteine (+57.02146)
**Variable modifications**	Oxidation (M), Acetyl (Protein N-term)
**Mass tolerance for precursor ions**	20 ppm for first search (before recalibration) and 4.5 ppm for main search (after recalibration)
**Mass tolerance for fragment ions**	0.5 Da
**False Discovery Rates at Peptide and Protein levels**	1 % at both peptide and protein level
